# 盐酸埃克替尼治疗*EGFR*突变状态明确的晚期非小细胞肺癌的临床观察

**DOI:** 10.3779/j.issn.1009-3419.2015.12.04

**Published:** 2015-12-20

**Authors:** 曦 李, 娜 秦, 敬慧 王, 新杰 杨, 新勇 张, 嘉林 吕, 羽华 吴, 卉 张, 靖颖 农, 权 张, 树才 张

**Affiliations:** 101149 北京，首都医科大学附属北京胸科医院肿瘤内科 Department of Medical Oncology, Beijing Chest Hospital, Capital Medical University, Beijing 101149, China

**Keywords:** 肺肿瘤, 盐酸埃克替尼, *EGFR*突变状态, 治疗效果, Lung neoplasms, Icotinib hydrochloride, *EGFR* mutation, Treatment outcome

## Abstract

**背景与目的:**

盐酸埃克替尼（icotinib hydrochloride）是我国第一个具有自主知识产权的小分子靶向抗癌新药，与吉非替尼和厄洛替尼相比，在化学结构、分子作用机理、疗效等方面相似。本研究观察盐酸埃克替尼治疗表皮生长因子受体突变状态明确的晚期非小细胞肺癌（non-small cell lung cancer, NSCLC）的疗效和毒副反应。

**方法:**

回顾性分析2009年3月-2014年12月间北京胸科医院收治的晚期NSCLC患者，表皮生长因子受体（epidermal growth factor receptor, *EGFR*）突变状态已知，均口服盐酸埃克替尼治疗，评价其疗效和毒副反应。

**结果:**

124例组织学证实的晚期NSCLC患者，其中*EGFR*突变型99例，野生型25例。全组客观有效率（ objective response rate, ORR）为51.6%，疾病控制率（disease control rate, DCR）为79.8%。突变型和野生型患者的ORR：63.6% *vs* 4.0%，DCR：93.9% *vs* 24.0%，两者均有统计学差异（*P* < 0.000, 1）。突变型和野生型患者的无进展生存期（progression-free survival, PFS）（分别为10.5个月和1.0个月）（*P* < 0.000, 1）。治疗相关的毒副反应主要为皮疹38例（30.6%），腹泻20例（16.1%）。

**结论:**

盐酸埃克替尼治疗EGFR突变的晚期NSCLC疗效肯定，耐受性好。

## Abstract (en)

**Background and objective:**

Icotinib is the first self-developed small molecular drug in China for targeted therapy of lung cancer. Compared to the other two commercially available epidermal growth factor receptor (EGFR) tyrosine kinase inhibitors, gefitinib and erlotinib, icotinib is similar to them in chemical structure, mechanism of activity and therapeutic effects. To explore the efficacy and side effects of icotinib hydrochloride in the treatment of the advanced non-small cell lung cancer (NSCLC) patients with *EGFR* mutation and wild-type.

**Methods:**

Patients with advanced NSCLC who were treated with icotinib hydrochloride in Beijing Chest Hospital were retrospective analyzed from March 2009 to December 2014.

**Results:**

The clinical data of 124 patients (99 with *EGFR* mutation and 25 with wild type) with advanced NSCLC were enrolled in this study. The patients' overall objective response rate (ORR) was 51.6 % and the disease control rate (DCR) was 79.8%; The patients with *EGFR* mutation, ORR was 63.6%, DCR was 93.9%. The ORR was 4.0% and the DCR was 24.0% in the wild-type patients. Median progression-free survival (PFS) with icotinib treatment in *EGFR* mutation patients was 10.5 months and 1.0 month in wild-type patients. The major adverse events were mild skin rash (30.6%) and diarrhea (16.1%).

**Conclusion:**

Monotherapy with icotinib hydrochloride is effective and tolerable for the advanced NSCLC *EGFR* mutation patients.

目前，肺癌的发病率和死亡率在世界范围内居癌症之首。据《中国2011年恶性肿瘤登记年报》报告，2011年我国肺癌发病率为48.32/10万，死亡率为39.27/10万。发病率和死亡率均居恶性肿瘤的首位^[1]^。其中80%-85%为非小细胞肺癌（non-small cell lung cancer, NSCLC），且多数患者确诊时已处于晚期（Ⅲb期或Ⅳ期）。化疗是晚期NSCLC的主要治疗手段，但疗效已达平台期。近年来，越来越多的与肺癌相关的驱动基因被发现，相关的靶向药物被应用于临床，小分子靶向治疗药物的出现对NSCLC的治疗具有里程碑性的意义，它使NSCLC的治疗进入了个体化治疗模式。吉非替尼及厄洛替尼现已广泛应用于晚期NSCLC的治疗，并取得较好的效果。盐酸埃克替尼是一种高效特异性的表皮生长因子受体（epidermal growth factor receptor, EGFR）酪氨酸激酶抑制剂（tyrosine kinase inhibitor, TKI），是我国第一个具有自主知识产权的小分子靶向抗癌新药，与吉非替尼和厄洛替尼相比，在化学结构、分子作用机理、疗效等方面类似，但具有更好的安全性，适用于晚期NSCLC患者的治疗^[2, 3]^。我们应用盐酸埃克替尼治疗124例*EGFR*突变明确的晚期NSCLC患者，并分析其临床疗效及药物相关的毒副反应。

## 1 材料与方法

### 1.1 临床资料

选取2009年3月-2014年12月首都医科大学附属北京胸科医院肿瘤科收治的晚期Ⅲb期或Ⅳ期NSCLC患者，所有患者均经组织学证实为NSCLC，具有完整资料。治疗前患者的血常规、肝肾功能及心功能均正常; 有可测量的临床病灶; 在治疗期间未同时行其他全身抗肿瘤治疗。

### 1.2 *EGFR*基因突变检测

全组患者均进行了肿瘤组织的*EGFR*基因突变检测，检测方法为扩增突变阻滞系统。*EGFR*基因检测时间均为一线治疗前。

### 1.3 治疗方法

全组患者均口服盐酸埃克替尼125 mg，3次/日，一直服用至疾病进展或毒副反应不能耐受为止。

### 1.4 评价标准

按照实体瘤疗效评价标准（Response Evaluation Criteria in Solid Tumors, RECIST）1.1版进行疗效判定，按照美国国立癌症研究所通用毒性标准4.0版评价毒副反应。疗效分为完全缓解（complete response, CR）、部分缓解（partial response, PR）、疾病稳定（stable disease, SD）和疾病进展（progressive disease, PD）。ORR包括CR和PR患者，DCR包括CR、PR和SD患者。

### 1.5 随访

124例患者均获得随访，通过门诊或电话随访获得患者的PFS，末次随访时间为2015年3月1日。PFS指患者自开始埃克替尼治疗到明确疾病进展的时间。

### 1.6 统计学方法

应用SPSS 22.0统计软件进行数据处理及统计分析，组间疗效和不良反应率比较应用χ^2^检验，*Kaplane-Meier*法分析患者的PFS，*P* < 0.05为差异有统计学意义。

## 2 结果

### 2.1 患者的一般特征

2009年3月-2014年12月我院肿瘤科收治的晚期NSCLC患者124例，所有患者均经组织学证实，具有完整随访资料。男性：55例，女性：69例。年龄36岁-80岁，中位年龄59.5岁。腺癌115例，其他病理类型9例。按2009年修订的肺癌临床分期标准，Ⅲb期：5例，Ⅳ期119例。有吸烟史45例。根据美国东部肿瘤协作组体能状态评分，0分-1分：83例; ≥2分：41例。

全组患者均进行了*EGFR*基因突变检测，其中19外显子缺失突变55例，21外显子L858R错义突变40例，18外显子突变1例，19外显子缺失突变和21外显子L858R点突变复合突变1例，20外显子S768I和21外显子L858R点突变复合突变2例，野生型25例。

其中盐酸埃克替尼用于一线治疗60例，维持治疗2例，二线及以上治疗62例。患者一般特征见表 1。

**1:** 124例患者的一般临床特征 Baseline characteristics of the study population (*n*=124)

Variables	Case (*n*=124)	*EGFR* mutation (*n*=99)	*EGFR* wild-type (*n*=25)
Age (year)			
< 70	105	82	23
≥70	19	17	2
Gender			
Male	55	38	17
Female	69	61	8
Smoking characteristics			
Yes	45	28	17
No	79	71	8
Performance status			
0-1	83	72	11
≥2	41	27	14
Histology			
Adenocarcinoma	115	96	19
Non-adenocarcinoma	9	3	6
Prior chemotherapy			
0	60	55	5
≥1	62	42	20
Maintenance therapy	2	2	0
EGFR: epidermal growth factor receptor.			

### 2.2 总体疗效

124例患者均服用盐酸埃克替尼1个月以上，服药1个月后评价疗效。全组124例患者中，PR 64例，SD 35例，PD 25例。ORR为51.6%，DCR为79.8%。*EGFR*突变患者的99例患者中，PR 63例，SD 30例，PD 6例。ORR为63.6%，DCR为93.9%。其中19外显子缺失突变患者的ORR为74.5%，DCR为94.5%;21外显子L858R突变患者的ORR为50.0%，DCR为95.0%。19外显子缺失突变与21外显子L858R突变患者相比，ORR差异有统计学意义（*P*=0.012），DCR差异无统计学意义（*P*=0.571）。18外显子突变1例，疗效PD; 19和21外显子复合突变1例，疗效PR，20和21外显子复合突变2例，疗效1例PR，1例SD。野生型患者25例，ORR为4.0%，DCR为24.0%。突变型与野生型的ORR及DCR均有统计学差异（*P* < 0.000, 1）（表 2）。

**2:** *EGFR*突变患者与野生型患者的疗效比较 The efficacy of icotinib in *EGFR* mutation and wild-type patients

Response	Mutation	Wild-type	*P*
Complete response	0 (0)	0 (0)	-
Partial response	63 (63.6)%	1 (4.0)%	-
Stable response	30 (30.3)%	5 (24.0)%	-
Progressive disease	6 (6.1)%	19 (72.0)%	-
Response rate	63.6%	4.0%	< 0.001
Disease control rate	93.9%	24.0%	< 0.001
Median progression-free survival (month)	10.5	1.0	< 0.001

已知突变阳性的99例患者中，一线治疗55例，其中PR 39例，SD 14例，PD 2例，ORR为70.9%，DCR为96.4%。2例患者用于维持治疗，均为PR，其中21外显子错义突变及20和21外显子同时突变各1例。二线及以上治疗42例，其中PR 22例，SD 16例，PD 4例，ORR为52.4%，DCR为90.5%;一线与复治患者的ORR及DCR均无统计学差异（*P*=0.061, *P*=0.233）。

全组的中位PFS为6.0个月。突变患者的中位PFS为10.5个月，野生型患者的中位PFS为1.0个月（*P* < 0.000, 1）（图 1A）。99例突变阳性患者疗效的单因素分析显示，各组均无统计学差异（表 3）。19外显子缺失突变患者的中位PFS为14.0个月，21外显子L858R突变的中位PFS为6.0个月，两者差异无统计学意义（*P*=0.185）（图 1B）。19外显子缺失和21外显子L858R复合突变的PFS尚未达到（已达14.2个月），20外显子S768I和21外显子L858R复合突变的PFS尚未达到（已达39.5个月），18外显子突变的PFS为1.0个月。突变阳性患者中，一线治疗的PFS为12.9个月，复治的PFS为7.5个月，两者差异无统计学意义（*P*=0.170）（图 1C）。

**1:**
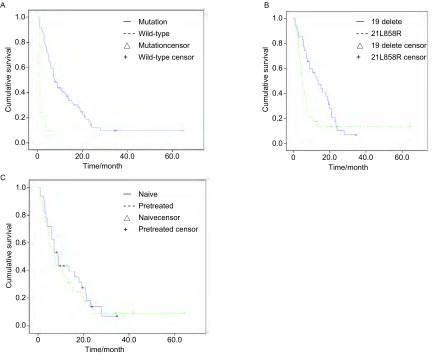
组间生存曲线的比较。A：*EGFR*突变型患者和野生型患者的PFS比较（10.5个月*vs* 1.0个月，*P* < 0.001）；B：*EGFR* 19外显子缺失突变与21外显子L858R突变患者的PFS比较（14.0个月*vs* 6.0个月，*P*=0.185）；C：*EGFR*突变患者中初治与复治的PFS比较（12.9个月*vs* 7.5个月，*P*=0.170） Comparison of survival curves between groups. A: PFS in *EGFR* mutation and wild-type patients (10.5 months *vs* 1.0 month, *P* < 0.001); B: PFS in *EGFR* exon 19 delete and exon 21 L858R mutation patients (14 months *vs* 6.0 months, *P*=0.185); C: PFS of *EGFR* mutation patients naive vs pretreated (12.9 months *vs* 7.5 months, *P*=0.170).

**3:** 99例*EGFR*突变患者的单因素分析 Univariate analysis of PFS in 99 mutation patients

Variables	PFS (months)	95%CI	*P*
Age (year)			0.545
< 70	8.7	4.6-12.7	
≥70	16.0	9.0-22.9	
Gender			0.845
Male	10.5	4.0-16.9	
Female	11.6	3.9-19.2	
Smoking characteristics			0.348
Yes	7.0	0-16.5	
No	10.8	5.3-16.3	
Performance status			0.051
0-1	10.6	2.4-16.8	
≥2	10.0	2.8-18.2	
Histology			0.427
Adenocarcinoma	10.8	5.5-16.1	
Non-adenocarcinoma	4.0	2.4-5.6	
Prior chemotherapy			0.170
0	12.9	5.2-20.6	
≥1	7.5	1.8-13.2	
PFS: progression-free survival.			

### 2.3 症状缓解情况

全组有70例（56.5%）患者在治疗后有不同程度的症状缓解，主要缓解的症状为咳嗽、喘憋、疼痛、声嘶等。多数患者在服药2周内出现肿瘤相关症状的缓解。

### 2.4 毒副反应

全组124例患者中，服用盐酸埃克替尼后出现的相关不良反应主要为皮疹38例（30.6%），其中Ⅰ度33例，Ⅱ度5例。腹泻20例（16.1%），其中Ⅰ度18例，Ⅱ度2例。转氨酶升高14例（11.3%），均为Ⅰ度，其他少见的不良反应为恶心、胃部不适、皮肤瘙痒、干燥、甲沟炎等。全组无一例患者因无法耐受毒副反应而停止治疗。

## 3 讨论

EURTAC^[4]^、OPTIMAL^[5]^、WJTOG3405^[6]^等多项大型的国际多中心随机对照临床研究均证实对于*EGFR*敏感突变的晚期NSCLC患者，EGFR-TKI治疗的PFS及ORR均优于传统细胞毒药物的化疗。盐酸埃克替尼作为第一代小分子的EGFR-TKI，在治疗晚期NSCLC方面，与吉非替尼和厄洛替尼相同，*EGFR*突变状态是其独立的疗效预测因素。ICOGEN研究^[7]^显示，埃克替尼和吉非替尼均可明显改善NSCLC患者的生活质量，且差异无统计学意义; 但埃克替尼总不良反应发生率低于吉非替尼。本组124例患者均已知*EGFR*突变状态，99例突变患者的ORR为63.6%，DCR为93.9%，中位PFS为10.5个月，与ICOGEN研究中埃克替尼治疗突变患者的亚组分析结果（ORR：62.1%，DCR：86.2%，中位PFS为7.8个月）相似。本研究中25例野生型患者选择埃克替尼治疗的原因是患者及家属拒绝化疗、PS评分无法耐受化疗或在*EGFR*基因检测结果尚未回报时已经开始治疗，ORR为4.0%，DCR为24.0%，PFS仅1.0个月，ICOGEN研究中吉非替尼及埃克替尼治疗*EGFR*野生型患者的ORR分别为：5.1%和3.8%，BR21研究^[8]^中，厄洛替尼治疗*EGFR*野生型患者的ORR为7.0%，结果均相似。在既往的临床研究中，有极少部分*EGFR*野生型患者应用EGFR-TKI治疗有效，可能与肿瘤异质性、检测的假阴性或化疗后*EGFR*突变状态的改变及可能同时存在其他少见的未知EGFR-TKI敏感性分子生物学机制有关。

19外显子缺失和21外显子L858R错义突变约占*EGFR*总体突变的85.0%，是EGFR-TKI的敏感突变^[9]^。WJTOG3405^[6]^、ICOGEN^[7]^及IPASS^[9]^研究结果显示19外显子缺失突变的患者接受EGFR-TKI治疗时疗效好于外显子21L858R突变的患者，本组得到同样结果，两组的ORR差异有统计学意义（*P*=0.012），虽然两组的PFS的差别未达到统计学差异，但19外显子缺失的PFS明显长于21外显子点突变的PFS（14.0个月*vs* 6.0个月）。Lee等^[10]^在2015年发表了一篇*meta*分析，关于化疗与EGFR-TKI一线治疗*EGFR*突变的晚期非小细胞肺癌的疗效比较，纳入了NEJ002、EURTAC、LUXLUNG6在内的8个临床研究，共计1, 649例患者，亚组分析结果显示：19外显子缺失突变较21外显子L858R突变有更长的PFS（11.8个月*vs* 10.0个月），差异有统计学意义（HR=1.39; 95%CI: 1.10-1.76; *P*=0.006），19外显子缺失突变人群更能从EGFR-TKI治疗中获益，可能对肿瘤信号传导通路的不同作用相关。韩国学者^[11]^对306例*EGFR*突变患者中复合突变的发生率及EGFR-TKI的疗效进行研究，结果显示复合突变的发生率在7.3%，19外显子缺失突变与21外显子L858R错义突变的复合突变疗效最好（ORR：74.8%，PFS：11.9个月），复合突变中包含有19外显子缺失突变或21外显子L858R错义突变者次之（ORR：68.8%，PFS：8.1个月）。日本的一项研究^[12]^显示复合突变发生率为9.0%，19外显子缺失突变与21外显子L858R错义突变的复合突变应用吉非替尼治疗ORR为86.0%，中位PFS达16.5个月。本研究中有3例患者为复合突变（3.0%），1例为19外显子缺失突变和21外显子L858R点突变，1例为20外显子S768I点突变和21外显子L858R点突变，两例疗效均为PR，PFS都超过10.0个月，1例20和21外显子复合突变的PFS为4.0个月，与日韩的研究结果相似。

既往多项研究证实针对*EGFR*突变人群，EGFR-TKI初治和复治的疗效相近，埃克替尼上市后的ISAFE临床研究^[13]^的亚组分析显示，在*EGFR*突变人群中，一线治疗与复治的ORR无统计学差异（*P*=0.215）。本组结果虽然一线与复治相比ORR、DCR及PFS均无统计学差异，但一线治疗的ORR及PFS均好于复治患者（70.9% *vs* 52.4%;12.9个月*vs* 7.5个月），考虑可能与本组*EGFR*突变检测均为一线治疗前，二线及二线以上患者未行疾病进展后的复测，*EGFR*基因的突变状态可能在化疗后及疾病进展过程中发生变化有关。本组针对已知突变人群的PFS的单因素分析显示，腺癌与非腺癌相比，PFS的差异较大（10.8个月*vs* 4.0个月），但尚未达到统计学差异，与非腺癌患者病例数较少相关，需要进一步进行相关研究。

INFORM研究^[14]^和SATURN研究^[15]^确立了EGFR-TKI在晚期NSCLC维持治疗中的地位。本研究中，一线化疗后维持治疗的2例患者，一线化疗的疗效均为SD，维持治疗的疗效为PR，其中1例为20外显子S768I点突变和21外显子L858R点突变，到随访结束PFS已经达到39.5个月，现仍服药中，提示埃克替尼在*EGFR*突变敏感人群中维持治疗有较好疗效，但病例数较少，仍需进一步研究证实。

本研究显示，盐酸埃克替尼治疗后，患者临床症状有所改善，症状缓解中位起效时间多为10 d-14 d，缓解程度较明显，主要缓解的症状为咳嗽、喘憋、疼痛和声嘶等。

本组盐酸埃克替尼的主要不良反应为皮疹和腹泻，发生率分别为：30.6%和16.1%，均为Ⅰ度和Ⅱ度。其他少见不良反应为转氨酶升高、恶心、轻度胃部不适、皮肤瘙痒、干燥和甲沟炎等，以上不良反应的发生率及严重程度与ICOGEN研究^[8]^及近期的研究^[16-18]^数据相似，其发生率及皮疹的严重程度均低于吉非替尼^[11]^，更低于厄洛替尼^[15]^，埃克替尼的不良反应较轻，耐受性较好。

综上所述，盐酸埃克替尼治疗*EGFR*敏感突变的晚期NSCLC疗效肯定，可应用于各线治疗及维持治疗。盐酸埃克替尼治疗后患者症状缓解快，缓解程度明显，治疗相关的毒副反应较轻，患者耐受性好。
